# A collaborative approach to improving patient access in general practice: impact of three different pilot schemes in 12 general practices in Greenwich

**DOI:** 10.1080/17571472.2016.1173946

**Published:** 2016-06-10

**Authors:** Melanie Lawless, Ellen Wright, Jackie Davidson

**Affiliations:** ^a^ NHS Greenwich Clinical Commissioning Group, London, UK; ^b^ Department of Primary Care and Public Health Sciences, King’s College, London, UK; ^c^ Public Health, Royal Borough of Greenwich, London, UK

**Keywords:** Patient access, managing demand, GP telephone triage, online consultations, practice workload

## Abstract

**Background:**

With rising patient demand and expectations, many practices are struggling to respond to the demand for appointments.

**Objective:**

To investigate different approaches to improving access to general practice and assess the impact on (i) patient experience, (ii) practice staff experience and (iii) activity in A&E and walk-in centres.

**Method:**

Greenwich CCG piloted three approaches in 12 volunteer practices. The schemes were:

(1) Systematic GP telephone triage of all appointment requests.
(2) Analysis and comparison of practice data including demand and capacity to identify opportunities for improvement.
(3) Online consultations.

Qualitative and quantitative evaluation was undertaken.

**Results:**

Overall results were inconclusive and no one pilot scheme was overwhelmingly successful in improving patient experience of access or reducing practice workload.

Scheme 1 telephone triage: In some cases, overall demand on clinician time through the day reduced as face-to-face consultations were replaced with shorter telephone consultations. However, in other practices, total consulting time went up when telephone consultations took longer than the suggested average 5 min.

Scheme 2 practice analysis and benchmarking: The pilot practices implemented no significant changes.

Scheme 3 online consultations: Take up was low, with users as a percentage of total list size dropping significantly to even lower levels in the second half of the pilot – from 3.13% in the first three months to 1.20% in the second three months.

**Conclusion:**

As the pilots did not improve the overall patient experience of access or practice workload, the pilot schemes were not rolled out by the CCG. From the CCG’s point of view, it was valuable to test out the effect of a scheme before committing further resources.

## Key messages

• Telephone triage does not consistently result in improved access.
• Knowing what needs to change in order to improve access is not enough – putting the changes into effect requires resources in terms of time and staff which are in short supply in general practice.
• Online consultations have a very low uptake and therefore limited effect on access.
• The impact of the above approaches on access can vary in different practices.

## Why this matters to me

**GP view – Ellen Wright:** As a GP in my own practice, I am only too aware of patient demand and the struggle we have to meet it. As Chair of the CCG, at every meeting we hold in public, I hear how people have difficulty getting an appointment with a GP and that this seems to be more difficult at some practices than others. I am also aware of GP recruitment difficulties and also the pressure on our local A+E service and that many patients say they attend because they cannot get a timely GP appointment.

I was aware of various ‘access initiatives’ – particularly the concept of telephone triage as a way of dealing with demand in a more systematic way and also use of online consultations to increase GP capacity – however, I also knew many GP colleagues were sceptical of the ability of these new models to really improve access and/or reduce workload. In addition, practices in Greenwich vary greatly across the borough in terms of size, premises, staffing, administrative systems and practice population. It seemed important to test some of these models first by the CCG offering to pilot them in selected practices.

The results, whilst perhaps not as clearly ‘successful’ in terms of meeting the objectives as we would have liked, have generated rich learning and an insight into the pressures on practices and also a willingness of the pilot practices to share their experiences with each other. It has also resulted in a comprehensive ‘good access handbook’ for practices which has incorporated the learning from all three schemes and contains practical suggestions for improving access developed from them. This handbook has been made available to all practices.

**Public Health view – Jackie Davidson:** Improving population-level health outcomes, including tackling health inequalities, not only hinges upon health improvement and health protection, but also on the quality and accessibility of NHS health care services and whilst life expectancy is improving for the wider population, there remains significant differences with those most vulnerable or deprived having poorer outcomes across a range of indicators compared to the general population. This is true of access to health care.

Locally, data highlight significant variations across practices in patient satisfaction with GP access. Nationally, evidence suggests that patients of practices serving the most (compared to the least) deprived areas are less satisfied with GP access and more likely to attend A&E.[1] We know that for every 100 attempts resulting in a GP consultation, there are 1.67 attempts that result in a visit to A&E and this equates to 5.77 million A&E attendances nationally that are preceded by an inability to get a suitable appointment.[2] Whilst there is no guarantee that, even if GP access is improved, the same patients would be deterred from attending A&E, there remains a need for more studies to explore the best way to improve access.

The role of primary care is pivotal as it acts as a gatekeeper to the health system and is the main point of contact with the patient and action needs to be taken to improve it, at the same time as resolving confusion around GP registration and eligibility. This pilot has provided valuable insight into the challenges that lay ahead to improve GP access.

## Introduction

General practice is facing unprecedented challenges. Increasing workload, rising numbers of patients with complex and multiple long-term conditions, a shift from hospital- to community-based care, an ageing population and growing patient expectations have placed unparalleled pressures on primary care in recent years.[3–5] Additionally, patients are becoming increasingly less satisfied with GP services with only 71% of Greenwich patients reporting that they were satisfied in 2014, the lowest reported level since the British Social Attitudes Survey began in 2009.[6] In Greenwich, patients experienced particularly low levels of satisfaction in relation to accessing GP appointments, although this did vary significantly between practices.[7]

In 2013, with the support of Greenwich CCG, a number of Greenwich practices came together to inquire into what was affecting access for their patients, to get a better shared understanding of the impact of demand and capacity and to test what models might help improve access. The CCG commissioned three different schemes aimed at improving GP access, firstly GP-led telephone triage, secondly, individualised improvements through demand/capacity analysis and thirdly, online consultations. The evaluations of these pilots are presented in this report. Throughout the pilot process, the practices came together to share the learning and identify the next steps for the individual practices.

## Aim and objectives

The programme aimed to improve access to general practice with three specific outcomes: (1) improved patient experience of access to general practice, (2) reduced workload of GP/practice staff through improved demand and capacity management and (3) reduced A&E and walk-in attendances.

## Methods

### Intervention: the three access schemes

#### Access Scheme 1: systematic, GP-led telephone triage of all patient requests for appointments, telephone consultations or visits

Five practices participated in Scheme 1. The GP-led telephone triage scheme involved receptionists advising all patients telephoning for an appointment that a GP would call back soon. Consequently, a GP telephoned and either (1) offered telephone consultation or (2) booked the patient into a same day, face-to-face appointment. An external provider undertook a detailed analysis of a practice’s supply and demand needs and provided practice-based training and ongoing support for the new system.

#### Access Scheme 2: identifying opportunities for improvement using a tool for analysing practice demand and capacity

Five practices participated in Scheme 2. Using a web-based demand/capacity tool, an external provider analysed the current demand/capacity of a practice, benchmarked this against participating practices and offered suggestions for improvement. Practice information was triangulated using data from the GP patient survey. All practices attended an individual practice meeting to discuss the findings and an off-site workshop with other Scheme 2 practices. Practices initiated improvements as they deemed fit.

#### Access Scheme 3: patient online consultations

Three practices participated in Scheme 3. The CCG piloted an online self-help advice and GP consultation facility. The model encouraged patients to consider self-help and other services first, moving on to a consultation with a GP in their practice after other avenues had been explored (including pharmacist and NHS 111 advice). The Scheme aimed to improve productivity within the practice as well as improve access.

An external provider supported this pilot and set up the online system on the pilot practice website. This enabled patients to go online for conditions or concerns that were not urgent, including access to a self-diagnosis algorithm. Each practice was encouraged to develop and deliver a marketing plan to ensure that their patients were aware that this facility was available.

### Research methods

The evaluation consisted of four aspects:

(1) Tracking measures for each pilot scheme by each provider. Measures included:
  • Scheme 1 – average days’ wait to see a GP; number of consultations and appointment type.
  • Scheme 2 – telephone demand by hour of day; average call length; availability of appointments; appointment type; consultation rate; and skill mix.
  • Scheme 3 – total number of unique visitors to the site during the reporting period; number of users who accessed the condition self-help pages; number of users who accessed the pharmacy-related self-help pages; number of completed clinician call back requests; and number of completed e-consults.
(2) Securing quantitative and qualitative evidence of the impact of the different schemes on patients and staff. This included:
  • A pre- and post-pilot survey of practice users.
  • In-depth interviews with GPs, practice managers/receptionists and patients.
(3) Analysing the impact of the schemes on A&E attendances/Walk-in Centres (using SUS data 2013/14 and 2014/15) and on practice workload for Scheme 1 (using data gathered online by the provider supporting Scheme 1 practices).
(4) Identifying lessons learned. These were gathered from two workshops established to share progress and explore common problems being encountered by participating practices.

#### Quantitative survey methodology

The study used a convenience sample of GP patients as this provided a quick and easy method with high levels of completion rates. As a consequence, the results are not representative of all GP practice populations and since different patients were contacted pre- and post-intervention, the results are not indicative of any real change in individual patients.

A sample size of 535 and 1277 (427 Scheme 1, 425 Scheme 2 and 425 Scheme 3) was used in the baseline and post-intervention surveys, respectively. Statistically significant findings were reported at the 95% confidence level.

#### Qualitative survey methodology

Telephone interviews and face-to-face in-depth semi-structured interviews were conducted with patients and staff. Twenty-one patients were interviewed in total: 8 from Scheme 1, 7 from Scheme 2 and 4 from Scheme 3. The interviewees were selected based on their experience of using the schemes and recruited through the post-pilot survey.

A GP, practice manager and/or other member of staff from each participating surgery were interviewed (21 staff in total). To allow interviewees to speak candidly about their experiences, these interviews were recorded anonymously, with the scheme identified but not the individual practices.

## Results

### Impact on objective 1: patient experience

#### Pilot Scheme 1 patient experience

**Figure 1.**
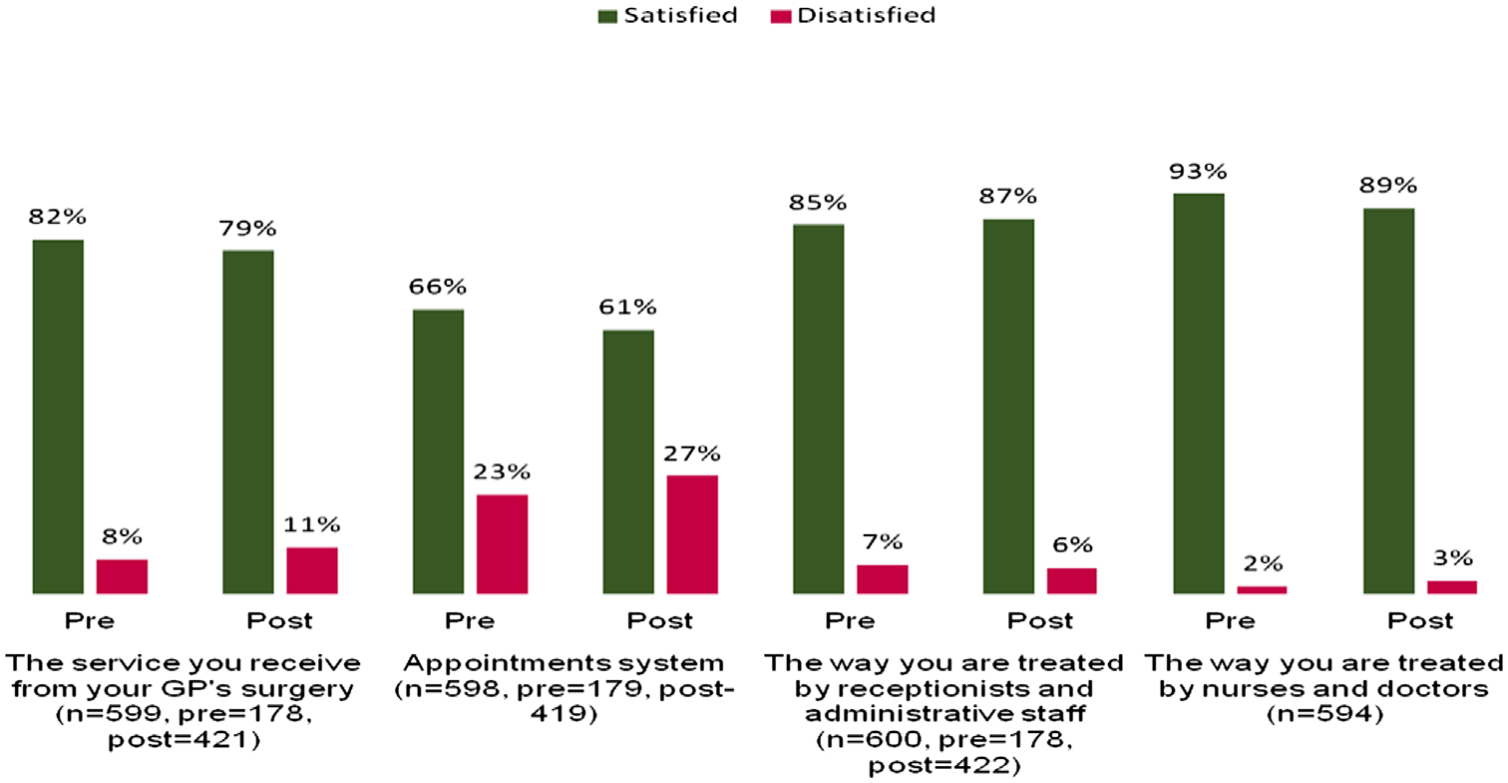
Average patient satisfaction for Scheme 1.

**Figure 2.**
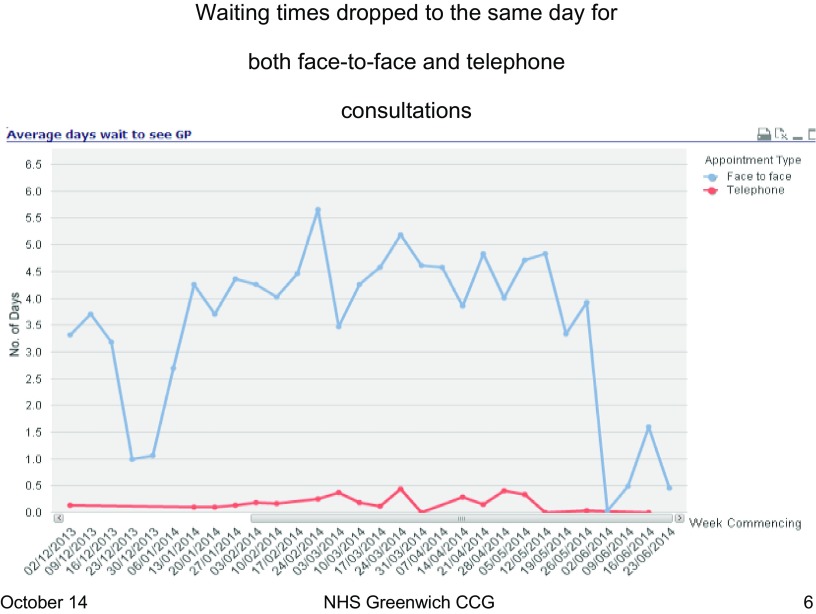
Average patient waiting times for Scheme 1 practice A.

There was no statistically significant change in *patient satisfaction* levels *overall or with the appointment systems* in Scheme 1 practices (Figure 1). This was despite the fact that waiting times for both telephone and face-to-face consultations reduced from four days to less than a day (Figure 2).

Three-quarters of patients were satisfied with phone consultations, although there was a significant degree of polarisation with older patients being less likely to be satisfied and the under 35s more likely to record an overall *improvement in the appointment system* (10% higher). Patients with positive experiences described being able to talk to a GP quickly without them having to visit a surgery. However, some patients had a general antipathy to phone consultations in principle and in practice: they were uncomfortable with the change, unfamiliar with phone consultations in general, felt disrupted by the lack of advance appointments and were concerned that the consultations were less thorough, although these patients were in the minority. There was no evidence that this was linked to people with limited English language skills, but there was evidence that it was older people who were more likely to have reservations.

Overall, most patients liked telephone consultations if they were offered as a choice. Patients learnt to ring at different times of the day and the frustrating ‘everyone to phone in at 8am’ effect was removed.

However, whilst patients described positive experiences of the scheme, in particular, the ability to speak to a doctor to resolve their problem in an efficient manner, a number of problems were raised. These problems fitted into three categories: the time it took to get through, the preference for booking appointments and unfamiliarity and lack of comfort with phone consultations in general.

#### Pilot Scheme 2 patient experience

**Figure 3.**
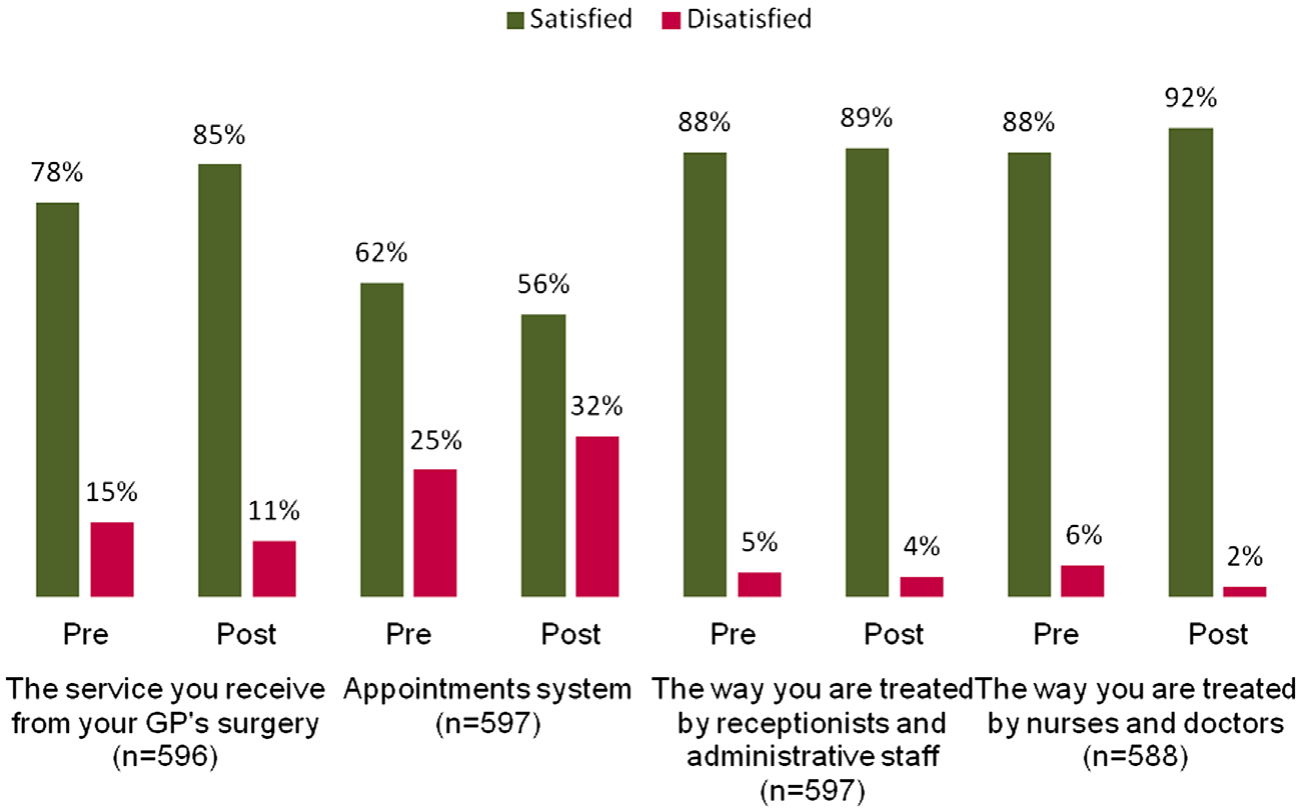
Average patient satisfaction for Scheme 2.

In Scheme 2, there was a statistically significant increase of 7% in the overall patient satisfaction with the GP service, but a significant drop in satisfaction with the appointment system of 6% (Figure 3). With the available data that do not take confounding factors into account, we cannot say that the changes seen were due to the pilot. In fact, the practice that reported the most marked net improvement reported that they had made minimal changes in the qualitative study.

Patients’ experiences of Scheme 2 did not have common themes as the participating practices had different processes in place and different priorities for improvement. Patients described challenges waiting to get appointments in some Scheme 2 practices, with waits of up to three weeks for appointments and waits on the phone of 20 minutes.

#### Pilot Scheme 3 patient experience

**Figure 4.**
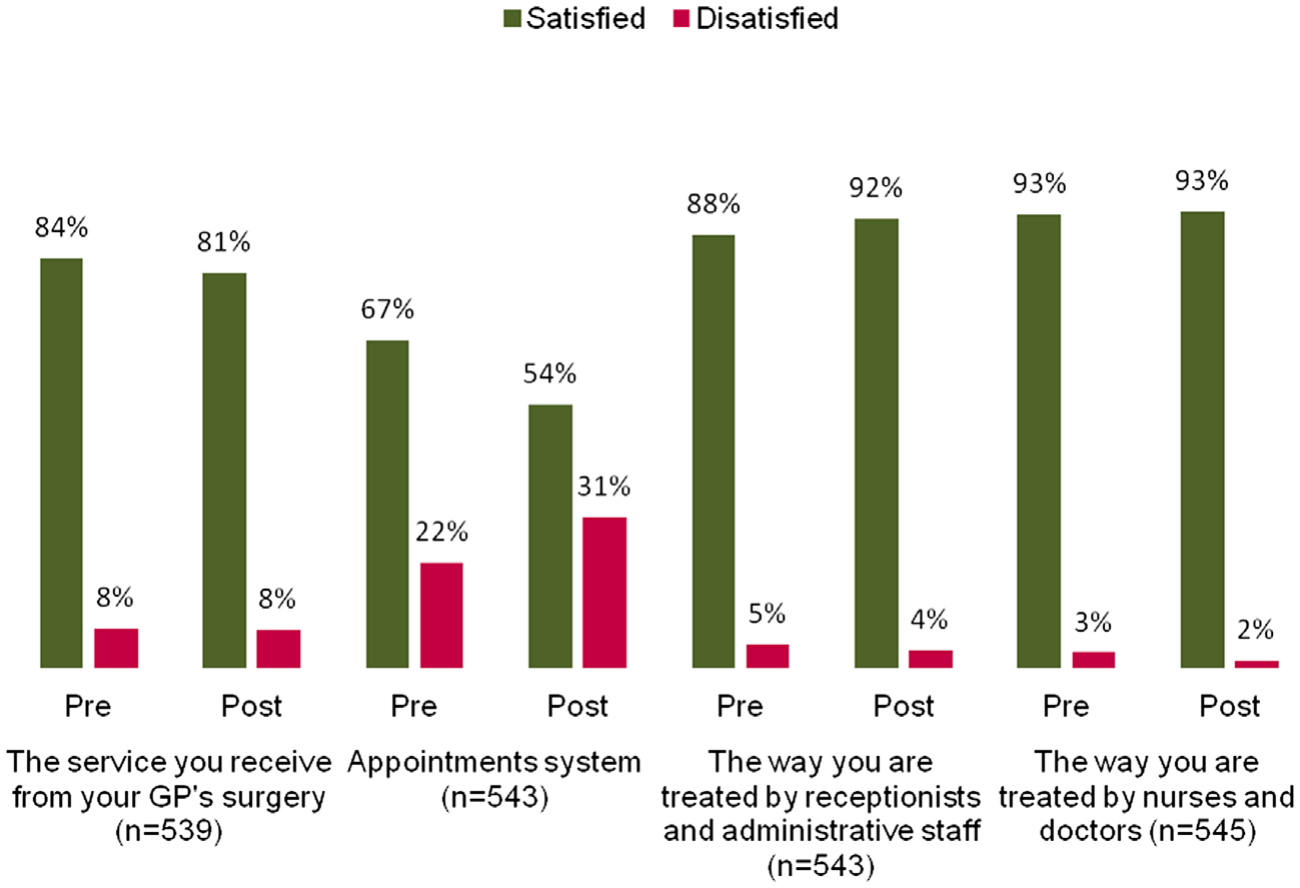
Average patient satisfaction for Scheme 3.

In Scheme 3, there was a 3% drop in the overall satisfaction with the GP service, although this was not statistically significant. There was, however, a 13% drop in patient satisfaction overall with the appointment system that was statistically significant (Figure 4). Since activity with the system dropped off considerably, it was difficult to find sufficient people who had used the system in this survey (49 patients were interviewed who had used it).

**Figure 5.**
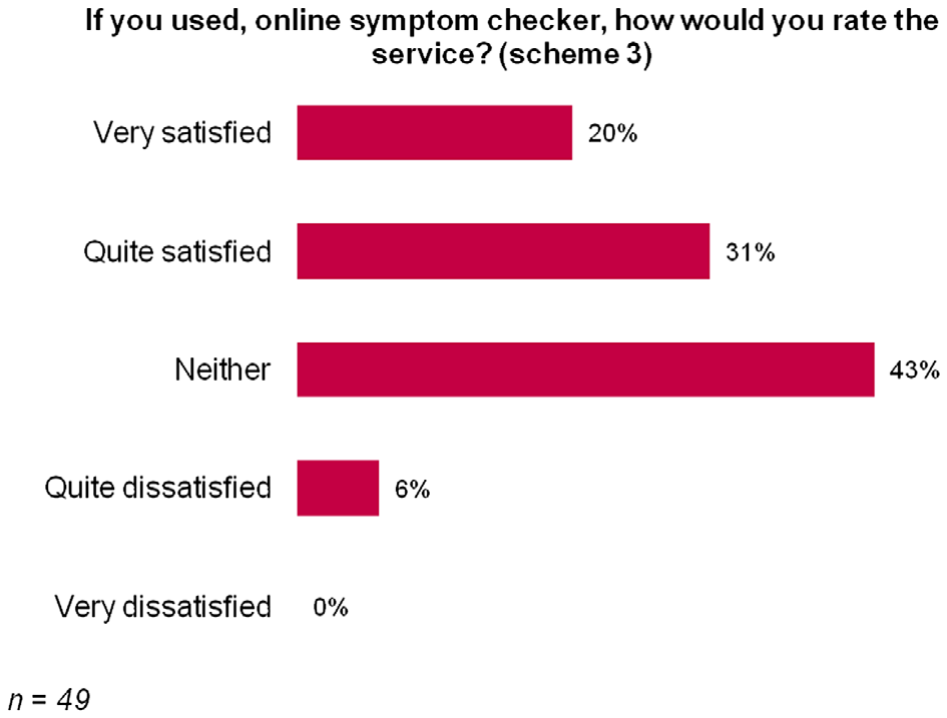
Perceptions of the online symptoms checker.

Out of those who used the online symptom checker, 51% were satisfied with the experience and 6% were dissatisfied (Figure 5).

## Impact on objective 2: practice staff views and workload

### Pilot Scheme 1 staff views and workload

**Figure 6.**
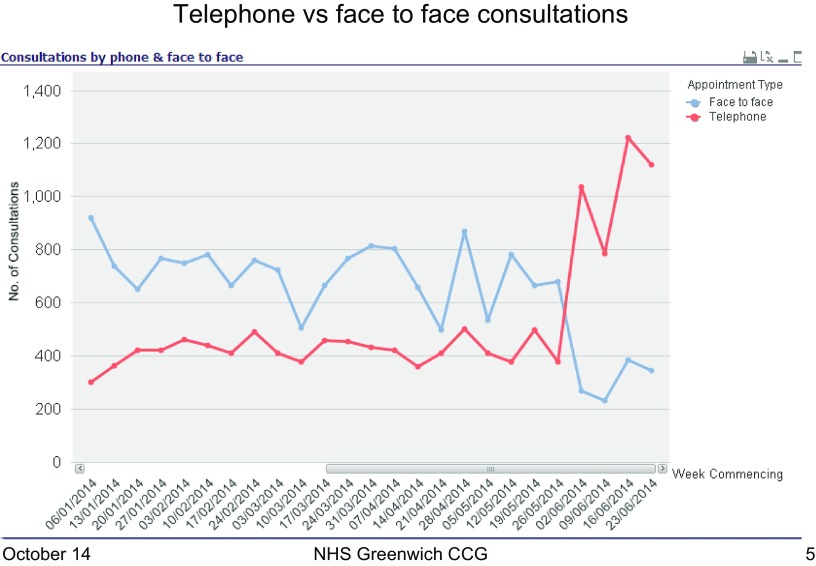
Average telephone vs. face-to-face consultations for Scheme 1 practice A (scheme implemented week beginning 12/05/14).

Practice staff felt Scheme 1 had a positive impact on same day patient access. This was evidenced also by the analysis that illustrated a reduction in waiting times for an appointment as face-to-face consultations dropped and telephone consultations rose (Figure 6). One practice reported that they were able to treat almost double the patients on a daily basis as well as enabling GPs to manage their time better.

For other practices, however, the new system created challenges and resulted in an increased workload for GPs. This was largely due to telephone consultations increasing at a greater rate than the corresponding reduction in face-to-face appointments compounded by the fact that telephone consultations were actually taking 8 min rather than the intended 5 min as prescribed by the scheme. If the latter had happened, then workload might have decreased.

A number of other issues impacted the effectiveness of Scheme 1:

• A number of practices encountered problems with their phone systems including a shortage of phone lines and problems with headsets. Practices that increased their phone capacity and increased staffing at key times were able to better manage the increased demand. A key piece of technology was a high-quality headset to help staff accurately hear patients and ensure their hands were free.
• Some GPs missed face-to-face contact with patients and felt opportunities for opportunistic health interventions were reduced.
• Some GPs were concerned that there might not be enough of a safety net for follow-ups.
• A significant number of patients complained they did not like being called back at work or on public transport.
• Getting locum cover GPs skilled and comfortable with telephone triage and telephone consultation was a challenge.
• The system was not suitable for trainee GPs as they needed experience of face-to-face consulting.

Practices’ plans to continue with Scheme 1 varied. Two practices are continuing with the telephone triage system with adaptations, two will not continue and one will be offering the choice of telephone consultations as an alternative to face-to-face appointments but allowing the same length of time (10 min) as for a face-to-face consultation. This practice also plans to address a gap in capacity through improving skill mix.

### Pilot Scheme 2 staff views and workload

In this scheme, only baseline demand/capacity data were collected; therefore, no comparative workload analysis was undertaken. Staff did not, however, perceive a decrease in workload.

Scheme 2 had varied impacts on the practices involved. In some, there were improvements, such as telephone triage and better receptionist screening in a way that had an impact on patient outcomes. For other practices, the recommendations were thought not to have added any new thinking and were only implemented to a limited extent. Once the recommendations had been presented, it was up to the individual practices to implement the suggested changes. The majority of practices felt that they were under a lot of pressure and to make changes, they needed specific change management support which was not part of the pilot.

**Figure 7.**
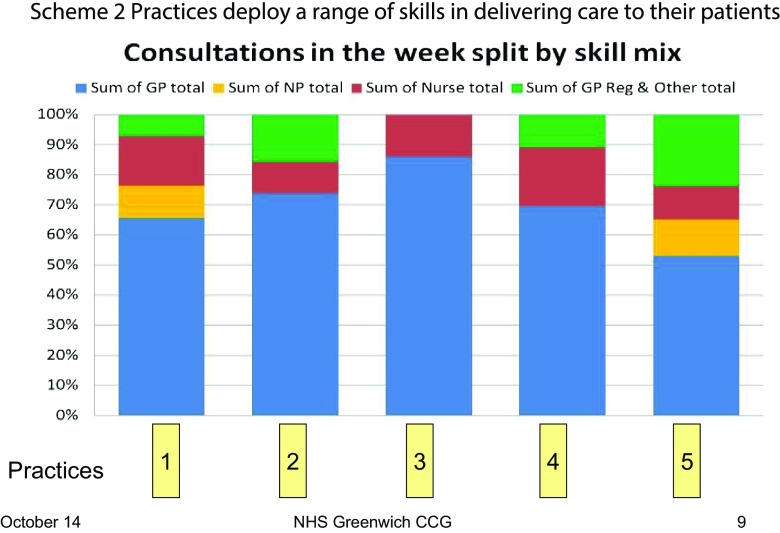
Variation in skill mix used by Scheme 2 practices.

Comparison between the practices revealed variation in the way practices operated and dealt with patient demand. For example, there was variation in how skill mix was deployed by the practices to meet patient demand as illustrated below (Figure 7).

NP = nurse practitioner; GP Reg = general practitioner registrar

#### Pilot Scheme 3 staff views and workload

Uptake of the online consultation facility, as measured by online activity by patients, was generally low when measured as a proportion of list size and ranged from 1.7 to 4.8% of total practice list size. There was a marked drop off in activity after the first three months (Table 1).

**Table 1.** Online consultations over the six months of the pilot (1.6.14 to 30.11.14).

Practice	Users	Self-help	Pharmacy self-help	LAS nurse call backs	GP consultations (plus as % of users)	Total users as % of list size
Practice 1 (pop 24,396)						
First 3 mths	901	187	66	14	25 (2.8%)	
					
Second 3 mths	288	51	18	9	21 (7.3%)	
					
Total	1172	238	83	23	46 (3.9%)	4.8%
						
Practice 2 (pop 9,979)						
First 3 mths	287	60	18	8	24 (8.4%)	
					
Second 3 mths						
	142	26	10	6	11 (7.7%)	
Total	420	81	28	14	35 (8.3%)	4.2%
						
Practice 3 (pop 4,626)						
First 3 mths	37	6	6	0	1 (2.7%)	
					
Second 3 mths						
	39	8	7	0	2 (5.1%)	
Total	76	14	13	0	3 (3.9%)	1.6%

*Users:* total number of unique visitors to the site during the reporting period.

*Self-help:* number of users who accessed the condition self-help pages.

*Pharmacy self-help:* number of users who accessed the pharmacy-related self-help pages.

*LAS nurse call backs:* number of completed clinician call back requests.

*Consultations completed:* number of completed e-consults.

Staff felt that whilst there may have been some positive impact, it was too small to detect. This pilot did not require the collection of demand/capacity data; therefore, there has been no analysis of workload.

### **Summary Staff views and workload**

Staff felt Scheme 1 had a positive impact on patient access but had created challenges in terms of workload, resulting in long sessions for the GPs and increased workload. This was supported by quantitative evidence of workload increases. Practice staff in the other schemes identified no improvement in workload.

## Impact on objective 3 A&E activity and walk-in centre activity

**Figure 8.**
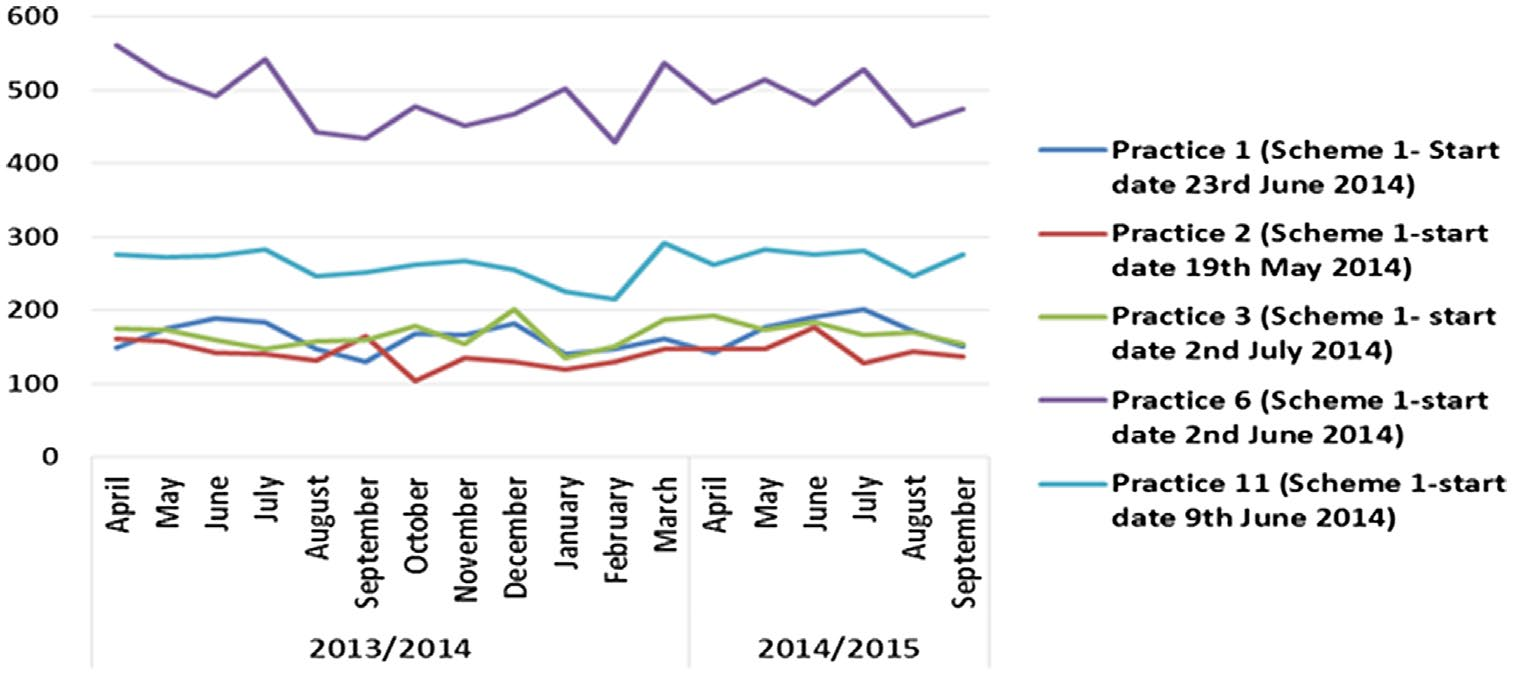
Scheme 1 access pilots impact on A&E activity.

Overall, the schemes did not have any significant impact on A&E or walk-in centre activity. Some practices in Scheme 1 noticed an initial decrease in A&E activity, but this was not sustained (see Figure 8) and possibly more likely linked to seasonal variation or natural fluctuation. There was no noticeable impact on A&E data from practices in Scheme 2. There was also no noticeable change in A&E data from the practices in Scheme 3 other than one practice that did experience a decline in A&E attendances, but again this was temporary.

**Figure 9.**
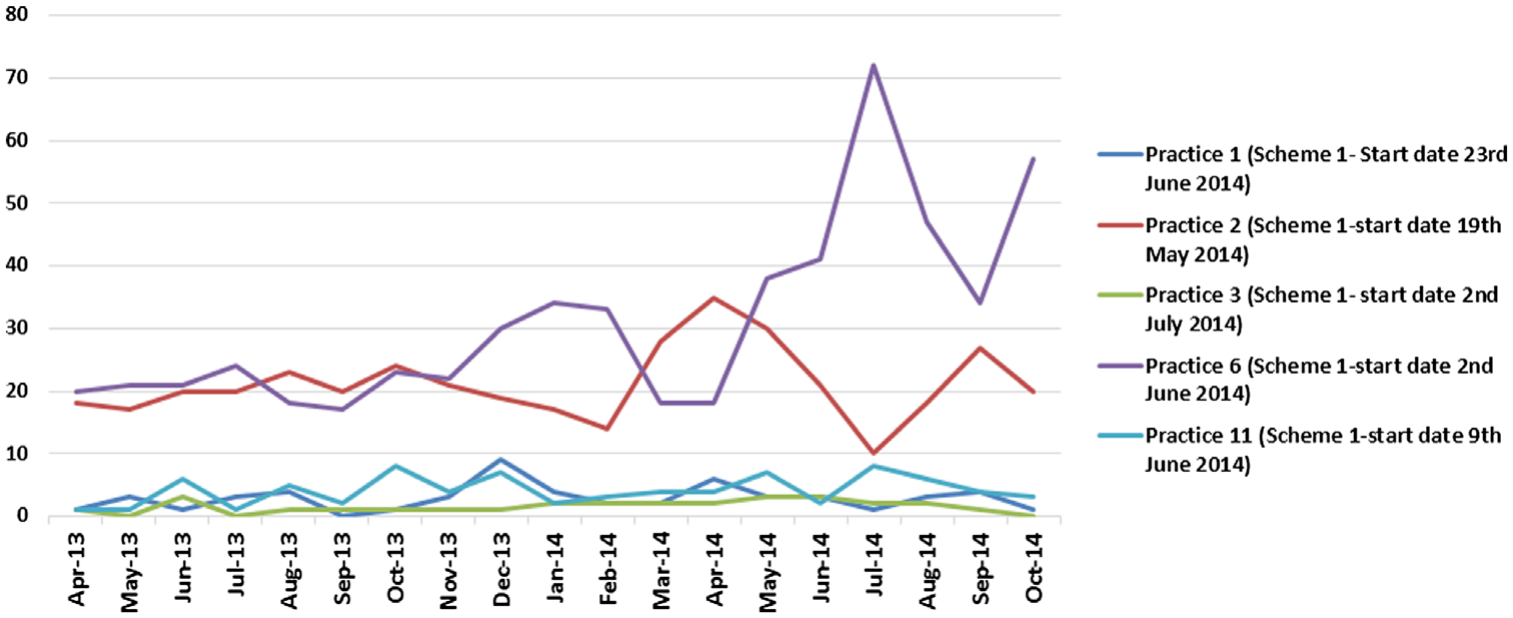
Scheme 1 pilot’s impact on activity at Clover Walk-in Centre attendances.

Similarly, there was no marked impact on walk-in centre activity for any of the schemes. In some cases, activity for Scheme 1 practices appeared to rise, but this appears to have been part of a trend that had begun prior to the pilots (see Figure 9).

## Discussion

This multi-method collaborative inquiry shows how clusters of practices can inquire and learn together, and also demonstrates the role the CCG and public health colleagues can take in supporting the process and measuring the outcomes.

### Demand and capacity

Patient demand for GP consultations, estimated to be an average of 6–7% of the practice list per week, is largely predictable. The variation (of between 5 and 10%) is driven by the practice deprivation and age profile. Within the week, there is a daily pattern, for example, Mondays are likely to be nearly a third of the total weekly demand, and this varies little between practices. The key for practices is to understand not just the demand for urgent or same day care, but total demand and to be aware that daily activity is not the same as daily demand.[8] Where there tends to be great variation, however, is in how practices match their capacity to meet their demand.

Capacity pressures were a constant feature of the practices involved in these schemes. Staff are pessimistic about system changes that are not matched by funding changes. Lack of capacity, head space, time and support prevent many general practices implementing improvements to the quality of their services, and redesigning the systems they work within, so that they end up working even harder rather than in a different, smarter way. Many practice staff know ‘what’ needs to be changed, but then struggle with ‘how’ it can be changed, knowing the constraints they are under. It is significant that although all of the participating practices received support and input from external providers (funded by the CCG), all said they would have liked more hands-on support to implement the recommended improvements.

An example of this is illustrated by the Scheme 3 pilot practices. New technology in primary care, including online consultations, is being promoted based on the view that patients who self-manage or contact 111 clinicians rather than coming in to the practice will reduce GP workload and free up appointments. Spending time on marketing and promoting the uptake of the available online consultation facility provided for them on their websites could have resulted in released productivity for practices. However, this did not happen.

The benchmarking exercise between the Scheme 2 practices revealed a lot of variation in the way practices operate and how they meet patient demand. There is scope in all practices to look at skill mix and develop the roles of a range of practice staff and work with other community-based professionals to share management of care and risk, e.g. with pharmacists.

The schemes also demonstrated variation in how patients respond to changes, with different levels of expectation in terms of waiting times and access to GP’s; waiting times are acceptable to some, but unacceptable to others. These different patient characteristics influenced the response to the changes implemented in the pilots.

### Study limitations

The study was a pragmatic evaluation of three pilot interventions and there are a number of limitations. Firstly, the satisfaction survey did not sample the same cohort of patients pre- and post-intervention and therefore did not capture the real change in patient’s experience as a result of the exposure to the intervention; thus, any changes in levels of satisfaction may have resulted from confounding factors such as differences in age groups interviewed, timing of survey and practice factors.

Secondly, the three schemes were being run in other parts of the country and were not designed specifically for Greenwich; thus, methodologies and data-sets were not uniform across all schemes making comparisons between schemes difficult.

Thirdly, the schemes were not implemented uniformly and the interventions were adapted. Within each scheme, there were significant discrepancies in the degree to which they were modified, varying levels of publicity raising awareness of changes and encouragement of uptake of new models.

In the case of Scheme 1, practices modified the scheme significantly. The impact of Scheme 2 was influenced by the voluntary nature of the recommendations with practices implementing them (or not) to a varying extent. Practices participating in Scheme 3 online consultations pursued different degrees of publicity to encourage uptake that had a significant bearing on the impact.

However, whilst we wanted to study the before and after impacts of the different models, we also wanted to encourage active learning as we went. The collaborative workshops we held during the pilots were valued by the practices as an opportunity to share both the successes and what was not working so well – and if necessary, adapt as we went.

## Conclusion

With all three schemes, the aim was to use the capacity available within a practice differently in order to release productivity as well as improve access. This was achieved in some practices but not in all, and not universally across any one pilot scheme. As a result, the CCG did not consider it to be good value for money to invest in rolling out any of the schemes across Greenwich.

GP workload was the crucial deciding factor in the particular model presented in Scheme 1 in whether practices wanted to continue with this approach post-pilot. The pilot worked best where practices had flexed and amended some aspects of the model. Some practices were able to successfully adopt the GP telephone triage model and make it work for them improving patient access and GP workload. Other practices demonstrated that when they implemented, the model workload increased. Therefore, it would appear that there would be little benefit in a universal roll out of this model.

There was a mixed response to Scheme 2, and a few improvements were made, but not enough to justify continued funding of this support to practices.

Although the uptake of online consultations in Scheme 3 was low, it was highlighted that marketing and promoting the availability of the facility by the practices were vital because patients would not use it if they did not know it existed. It was expected that use of the facility would grow over time as patients got to know about it. Instead, there was a drop off in activity over time – presumably, a reflection of initial marketing at the beginning of the pilot, but a reduction in marketing by the practice as time went on. In terms of patient satisfaction with the service offered, the majority of patients were either satisfied or neutral about the technology. This learning will be important to the CCG when considering any future online digital capability as technologies to improve patient access develop further.

Whilst not achieving the intended objectives, all the pilots helped us learn what elements of a general practice system contributed to improved access – what is key to getting capacity and demand into balance and what elements form barriers to good patient access.

What we have also learnt through this inquiry is how individual practices and practitioners vary in their response to access and what they value. Some GPs will work with the telephone triage system, even if it means more time is needed to see the waiting room clear at the end of the day and feel in control of the demand coming their way. Others prefer face-to-face contact with their patients that supports patient continuity and enables opportunistic lifestyle interactions.

Likewise, we have learnt how patient attitudes and responses impact access. An effective system of access in a practice must have the flexibility to offer patients and practitioners an approach that suits individuals as well as the majority. We also learnt the importance of communicating and engaging with patients and staff when changes are introduced. If patients do not understand the benefits of a new system, they are unlikely to embrace it.

This learning has been captured and described in the *Greenwich ‘Good Access Guide’* which contains practical advice and ‘top tips’ and has been disseminated to all practices in Greenwich.

The next stage of this inquiry into the challenge of improving patient access will be taken up by the GP provider networks that have been established in Greenwich. Working together at scale, with sharing of patient records already in place for the delivery of an extended long-term conditions contract, practices will identify together how best to share an approach to improving patient access in hours across a number of practices. The challenge will be how to keep the important aspects of flexibility and patient continuity. At the same time, the Greenwich Community Education Provider Network will identify, and provide, the support and skills practices need in order to improve access for patients.
